# Loot

**Published:** 1868-04-01

**Authors:** 


### *LOOT.*

---

***Blanching of the Hair.***

A paper read before the Royal Society, London, by Mr. Erasmus Wilson, has thrown new light on the question as to what causes the sudden whitening of the hair, often produced by fright or profound grief. He cites a case in which the hair was colored white and brown alternately from end to end. The white segments were about one-half the length of the brown, and the two together measured about one-third of a line. Under the microscope, the colors were reversed, and it was obvious that the opacity of the white portion was due to a vast accumulation of air globules packed closely together in the fibrous structure of the hair, as the medulla. There was no absence of pigment, but the accumulation of air globules veiled and obscured the normal color and structure. Mr. Wilson suggested the possibility of the brown portion being the day growth, and the white portion the night growth. He also said, in reference to the sudden blanching of the whole hair, of which there were many cases on record, that during the prevalence of a violent nervous shock the normal fluids of the hair might be drawn inward toward the body, in unison with the generally contracted and collapsed state of the surface; and that the vacuities left by this process of exhaustion might be suddenly filled with atmospheric air. An interesting discussion followed the reading of the paper. Dr. Sharkey alluded to a recent case of sudden blanching of the hair reported by Dr. Landois, of Griefswald, in Virchow's *Archiv.*, which was ascertained to be the result of an accumulation of air globules in the fibrous substance of the hair.

***Chemistry in Medical Jurisprudence.***

A man was tried for murder in the city of Dublin. The poison used, as charged, was *Hydrocyanic acid*, *Prussic acid*. The prosecution had all its own way, until the last moment, and the judge was charging the jury adversely to the prisoner. Christison, who was employed by the defence, had not arrived, in consequence of adverse winds delaying him on his passage from England to Ireland. This was before steamboats were known. At the critical moment we speak of, the toxicologist made his appearance in court. The judge, as a matter of justice to a man whose life was so critically and fearfully jeopardized, permitted the case to be reopened for the defence.

"My Lord," demanded Christison, "I respectfully request that you will place a portion of the saliva from your mouth on this plate."

His Lordship did as requested. Christison applied his tests to the saliva, and exhibited to the astonished judge, counsel and jury, precisely the exact results as the tests used by the experts for the prosecution in proving the presence of the poison in the supposed murdered person!

### *On the Production of Sexes.*

M. Coste has been led to doubt the truth of the hypothesis propounded by M. Thury, which supposes that every egg passes, during the period of its maturation, through two successive but continuous phases, during each of which it has a different sexual character. If fecundated in the first half, it would be a female; if in the latter, a male. From experiments on fowls, the author shows that the sexes are produced indifferently from eggs taken at the beginning, middle, or end of the laying. With regard to rabbits, M. Coste finds the same irregular results; in fact, altogether a larger number of males were born at the commencement of maturation. M. Thury's law is therefore not applicable to such mammals or to birds. The author is continuing his experiments to determine whether it holds good even in the bovine mammals, which M. Thury made the subject of his investigation.

### *A Simple Method of Protecting Water from the Action of Lead Pipe.*

*Dingler's Polytechnisches Journal* publishes a simple method, brought forward by Dr. Schwarz, of Breslau, for preventing the poisonous influence of lead pipes on water, by forming on the inside surface of the pipes an insoluble sulphuret of lead, which has proved so effective, that, after simple distillation, no trace of lead can be detected in water which has remained in the pipes for a long time. The operation, which is a very simple one, consists in filling the pipes with a warm and concentrated solution of *Sulphuret of potassium* or *sodium*. The solution is left in contact with the lead for about fifteen minutes. Commonly, a solution of *Sulphur* in *Caustic soda* will answer the purpose, and produce practically the same results. It is known that *Sulphuret of lead* is the most insoluble of all compounds of lead, and nature itself presents an example which justifies the theory of Dr. Schwarz, since water extracted from the mine of Galena does not contain lead, a fact which has occasioned surprise.

### *Neuralgia Mixture.*

Take *Quinia*, two drachms; tincture *Aconite*, two drachms; tincture *Gelsemin*, one drachm; *Ferri prussiate*, two drachms; neutralizing mixture, twelve ounces; simple syrup, twenty-five ounces. Mix. Dose, a teaspoonful every two or three hours.

### *To Remove Freckles.*

A French Journal recommends the following: Take *Napthaline*, ten parts; *Biphenate of soda*, one part; tincture of benzoin, cologne, each two thousand parts. Mix. A tablespoonful of this is to be added to a glass of cold water, four to eight fluid ounces, and the face then bathed with it every night and morning.

### *Ulceration of the Tonsils.*

Dr. G. W. Champ recommends the following wash as most effectual in ulceration of the tonsils, or apthous affections of the mouth: Take *pulv. Sulphate of zinc* and *Chlorate of potash*, of each, two drachms; strong sage tea, half a pint. Mix. Gargle the throat frequently.

